# HP1γ promotes the progression of colorectal cancer through interaction with RBPJ of the Notch signaling pathway

**DOI:** 10.1007/s10565-026-10205-z

**Published:** 2026-07-01

**Authors:** Rui-Bao Zhu, Yi-Jia Xie, Yu-Ting Han, Bo Peng, Wen-Ting Wang, Xin-Yi Lu, Yu-Tong Li, De-Cai Mao, Jin Sun, De-Xin Chen, Qin-Yun Che, Li Zheng, Dong-Lin Li, Feng-Rui Hou, Meng-Lin Mo, Shi-Yao Gong, Yi-Zhi Dong, Rong-Zhi Fu, Qing-Fei Liu, Fang-Lin Sun, Zhi-Jie Chang, Jian-Quan Ni, Ming-Yang Li

**Affiliations:** 1 School of Basic Medical Sciences, Tsinghua University, Haidian, Beijing, China; 2 SXMU-Tsinghua Collaborative Innovation Center for Frontier Medicine, Shanxi Medical University, Taiyuan, Shanxi China; 3 State Key Laboratory of Molecular Oncology, Tsinghua University, Haidian, Beijing, China; 4 Sichuan Academy of Giant Panda, Chengdu, Sichuan China; 5 Shandong First Medical, University&Shandong Academy Of Medical Sciences, Jinan, Shandong China; 6 Graduate School, Chinese PLA General Hospital, Haidian, Beijing, China; 7 Senior Department of Gastroenterology, The First Medical Center of Chinese, PLA General Hospital, Haidian, Beijing, 100853 China; 8 College of International Education, School of Beijing, University of Chemical Technology, Chaoyang, Beijing, China; 9 School of Pharmaceutical Sciences, Tsinghua University, Haidian, Beijing, China; 10 School of Life Sciences and Technology, Tongji University, Yangpu, Shanghai, China

**Keywords:** Colorectal cancer, HP1γ, RBPJ, Epigenetic dysregulation, Notch signaling pathway

## Abstract

Epigenetic dysregulation plays a pivotal role in cancer progression. Unlike HP1α and HP1β, which localize primarily to heterochromatin, HP1γ predominantly occupies euchromatin, suggesting a direct role in transcriptional regulation. Here, we identify HP1γ as a high-risk oncogene in colorectal cancer (CRC). Genome-wide analysis and disease-free survival (DFS) correlation studies revealed that high expression of HP1γ is associated with poor DFS in CRC patients, and we confirmed HP1γ upregulation in colon adenocarcinoma (COAD), the predominant CRC subtype. Functional assays demonstrated that HP1γ overexpression enhances tumor cell proliferation, migration, and colony formation *in vitro*, while siRNA-mediated knockdown suppressed these phenotypes. *In vivo* studies showed that HP1γ drives tumor growth, increasing both volume and weight in xenograft models. Mechanistically, HP1γ, via its chromo shadow domain (CSD), physically interacts with the beta-transducin repeat domain (BTD) of recombination signal binding protein for immunoglobulin kappa J region (RBPJ), the transcriptional factor of the Notch pathway. Intriguingly, RBPJ upregulation counteracts HP1γ-induced hyperproliferation, implicating HP1γ as a critical modulator of Notch signaling. Our findings establish HP1γ as a promoter of CRC progression through suppression of Notch pathway components, highlighting its potential as a diagnostic marker or therapeutic target in CRC.

**Supplementary Information:**

The online version contains supplementary material available at https://doi.org/10.1007/s10565-026-10205-z.

## Introduction

Colorectal cancer (CRC) ranks as the third deadliest malignant tumor worldwide, with colon adenocarcinoma (COAD) being the most common histological subtype of CRC. Despite progress in diagnosis and systemic treatment, most patients with metastatic CRC still die of their disease, indicating that the molecular mechanisms underlying the tumorigenesis and tumor growth of CRC remain largely elusive(Mármol et al. 2017; Dekker et al. 2019).

Cells within CRC have high molecular heterogeneity spatially(Sagaert et al. 2018). In addition to the microenvironment, intracellular signaling pathways play important roles in controlling cancer cell proliferation and differentiation. These pathways include Wnt, MAPK, PI3K, EGFR, TGF-β, and Notch signaling pathways. However, compared to other signaling pathways, the multifaceted Notch signaling has not been well studied in the CRC process(Fearon 2011; Malki et al. 2020; Nguyen et al. 2020).

As a well-conserved cell–cell communication route, the Notch signaling pathway mediates direct crosstalk between neighboring cells(Andersson et al. 2011). Signal-sending cells express Notch ligands including JAG1/2 and DLL1/3/4, which bind to Notch1–4 receptors on recipient cell(Henderson et al. 1994; Tax et al. 1994; Kopan and Ilagan 2009; Siebel and Lendahl 2017). Such binding induces conformational alterations that trigger successive proteolytic cleavage events, releasing the Notch intracellular domain (ICN)(De Strooper et al. 1999; Brou et al. 2000; Tian et al. 2008). The ICN then translocates into the nucleus and binds to the key transcriptional regulator RBPJ (Giaimo et al. 2021), leading to the exchange of corepressors with coactivators such as MAML1 and subsequent activation of downstream target genes(Jeffries et al. 2002; Wu et al. 2002). Studies over recent decades have revealed that Notch can either promote or suppress tumors depending on the tissue and cellular context(Aster et al. 2017), which indicates that the effect of Notch signaling is considerably diverse. For example, various Notch receptors show different changing trends in the tumor tissues of patients and are closely related to the development of cancer progression. Accumulating evidence has documented aberrant upregulation of Notch1 in human colorectal cancer (CRC). By contrast, Notch2 expression shows a significant inverse correlation with Notch1 levels in CRC, and reduced Notch2 expression serves as an independent predictor of poor prognosis in CRC patients. For Notch4, prior studies have established its downregulation in CRC tissues, and its mRNA level is validated as an independent prognostic biomarker for both disease-free survival (DFS) and overall survival (OS) in CRC patients(Aster et al. 2017; Shi et al. 2024). Collectively, due to the distinct roles of variable Notch receptors and complicated cross-talks with other signaling pathways, the regulation of the Notch pathway has not yet been fully studied, particularly on the transcriptional level coordinated by epigenetic factors(Wallberg et al. 2002; Liefke et al. 2010; Schwanbeck et al. 2011; Schwanbeck 2015; Skalska et al. 2015).

In addition to intrinsic factors, the microenvironment mediates the landscape of heterochromatin, contributing to the initiation and development of CRC(Punt et al. 2017; Kasprzak 2021). For example, loss of imprinting, which is involved in the silencing of the insulin-like growth factor II gene, will greatly increase the chance of CRC(Cui et al. 2003); however, as a key silencing factor, whether HP1 participates in tumor progression is not known. There are three HP1 variants in mammals: *CBX5* (*HP1α*), *CBX1* (*HP1β*), and *CBX3* (*HP1γ*)(Lomberk et al. 2006; Dialynas et al. 2008; Zeng et al. 2010). Unlike HP1α and HP1β, HP1γ is preferentially distributed in the euchromatin region(Kumar and Kono 2020; van Wijnen et al. 2021), indicating that HP1γ might directly interact with transcriptional factors to prevent gene transcription. However, there is little information about the role of HP1γ in CRC.

Here we performed bioinformatic analysis by using TCGA (The Cancer Genome Atlas) to predict high-risk genes in CRC. Interestingly, *HP1γ* is highly transcribed in CRC patients, and the high level of HP1γ causes a significant reduction in disease-free survival (DFS). In addition, HP1γ promotes cancer cell proliferation and migration *in vitro*, and the *in vivo* experiments also confirmed that HP1γ promotes tumor growth. Most interestingly, we found that HP1γ physically interacts with RBPJ, the key transcription regulator of Notch pathway. Overexpression of RBPJ can prevent the hyperproliferation of tumors triggered by high levels of HP1γ. Altogether, our study revealed that HP1γ is a fine regulator of the Notch pathway in CRC. Its requirement in the tumor growth enables it as a potential drug target for CRC treatment, and the ectopic expression enables it to be a marker of CRC for prognosis.

## Methods

### Ethical approval

This study strictly followed the Declaration of Helsinki. The use of archived human colorectal cancer and adjacent non-tumor tissues was approved by the Ethical Committee of Tsinghua University (IRB-00008273), with written informed consent from all enrolled patients. All in vivo animal protocols were approved by the Institutional Animal Care and Use Committee of Tsinghua University (No. 18-CZJ2), and performed in line with the ARRIVE guidelines for the ethical treatment of experimental animals.

### Construction of plasmids

The cDNA of HEK293T(RRID: CVCL_0063) was used as a template to amplify the CDS of HP1γ and RBPJ. To generate the transiently transfected plasmids, the pcDNA 3.1 (RRID: Addgene_12557) vector with HA or Flag tags was digested by *KpnI* and *XhoI* restriction endonucleases, and the CDS of HP1γ or RBPJ was cloned into the linearized vectors by Hieff Clone™ Plus One Step Cloning Kit. The sequences of primers were 5’-TTTAAACTTAAGCTTGGTACCGCCACCATGGCCTCCAACAAAACTAC-3’, 3’- GAAAGGTCCTCTAGACTCGAGTTGAGCTTCATCTTCTGGAC-5’ for HP1γ, 5’-TTTAAACTTAAGCTTGGTACCGCCACCATGGACCACACGGAGGGCTC-3’ and 3’-GAAAGGTCCTCTAGACTCGAGGGATACCACTGTGGCTGTAG-5’ for RBPJ, 5’-CTAGCTAGCATGGGATTTGCCAG-3’ and 3’- CCCAAGCTTTTACTTGTCATCGTC-5’ for HP1γ-CSD, 5’-CTAGCTAGCATGGTTAGTACCAGATACTTGCATGTAGAAG-3’ and 5’-CCCA AGCTTAAGCTTTTAAGCGTAATCTGGTACGTCGTATGGGTAACTAGATGTATACTCTGCCTTATCTGTGCTAATG-3’ for RBPJ-BTD.

The way to obtain the lentiviral donor vector is the same as above. The pCDH vector with EGFP select marker was digested by *XhoI* and *NotI* restriction endonuclease, and the pCDH vector with puro select marker was digested by *NheI* and *AsiSI* restriction endonuclease. The sequences of primers were 5’- ATAGAAGATTCTAGAGCTAGCGCCACCATGGCCTCCAACAAAACTAC-3’, 3’- CTGACGGGCACCGGAGCGATCGCTTACTTGTCATCGTCGTCCTTGTAATCTGATCCTTGAGCTTCATCTTCTGGAC-5’ for HP1γ and 5’- GTTCCAGATTACGCTCTCGAGGCCACCATGGACCACACGGAGGGCTC-3’, 3’-GGGAGGGAGAGGGGCGCGGCCGCTTAGGATACCACTGTGGCTG-5’ for RBPJ.

The Notch-responsive luciferase reporter plasmid employed in our experiments was gifted by Dr. Wei Wu from the School of Life Science, Tsinghua University.

### Small interfering RNA (siRNA) synthesis

siRNAs targeting HP1γ (si-HP1γ−1: 5'-GAUUGAAGCGUUUCUUAAC-3'; si-HP1γ−2: 5'-AGGAUUUGCCAGAGGUCUU-3') and negative control siRNA (si-NC: 5'-UUCUCCGAACGUGUCACGUTT-3') were synthesized by RiboBio (Guangzhou, China).

### Cell culture, transient transfection, and establishment of stable cell lines

HEK293T (RRID: CVCL_0063) cells were cultured in Dulbecco's Modified Eagle's Medium (Gibco, C11995500BT), human colon cancer cell line LoVo (RRID: CVCL_0399) was maintained in F12K (MACGENE, CM10025), human colon cancer cell line SW620 (RRID: CVCL_0547) was maintained in Leibovitz's L-15 (MACGENE, CM10045). All the media were supplemented with 10% fetal bovine serum (ExCellBio, FND500) and penicillin (100 units/ml)/streptomycin (100 units/ml) (Gibco, 15140122). The HEK293T cells and LoVo cells were grown at 37 °C with 5% CO_2_, and the SW620 cells were grown at 37 °C without CO_2_.

The transient cell transfections were performed by Vigofect reagent (Vigorous) according to the manufacturer's protocols. Stable cell lines were selected by puromycin or FACS after being infected with a specific lentivirus.

### Human colorectal samples and immunohistochemical staining

Clinical colorectal tissues were collected from patients at the Chinese PLA General Hospital. The study protocol was approved by the hospital’s Clinical Ethics Committee, with written informed consent from all participants. Specimen clinicopathological details are listed in Table 1. IHC staining was performed per established protocols, with primary antibodies including rabbit monoclonal anti-HP1γ (CST, Cat# 2619S, RRID: AB_2070984, 1:200) and rabbit polyclonal anti-Ki67 (Abcam, Cat# ab15580, RRID: AB_443209, 1:1000).

**Table 1:** The detailed information of clinical samples

Number	Gender	Age	TNM Stage	pStage	Degree of Differentiation
1	male	33	T1N0M0	I	High
2	male	69	T4N1M0	IIIB	low-mediate
3	male	62	T3N0M0	IIA	mediate
4	male	47	T3N0M0	IIA	mediate
5	female	68	T3N0M0	IIA	low-mediate
6	female	63	T3N2M0	IIIC	low-mediate
7	male	70	T4N1M0	IIIB	low-mediate
8	male	57	T3N0M0	IIA	mediate
9	female	81	T3N0M1	IV	mediate
10	female	81	T3N1M0	IIIA	mediate
11	male	43	T3N1bM0	IIIB	mediate
12	female	58	T3N0M0	IIA	mediate

### Western blot and immunoprecipitation

For immunoblotting analysis, freshly harvested cells or tissue samples were lysed in 2 × Loading buffer (5% SDS, 0.04% BPB, 20% glycerin, 10% Tris–HCl (pH = 6.8), 5% β-mercaptoethanol) and heat-denatured at 100 °C for 10 min. After electrophoresis on 12% SDS-PAGE gels, proteins were transferred onto nitrocellulose membranes. After blocking with 5% non-fat milk, the membranes were incubated with corresponding primary antibodies at 4 °C with overnight incubation. Primary antibodies, including mouse Anti-HA (Santa Cruz Cat#sc-7392, RRID: AB_627809, 1:1000), rabbit Anti-HP1 gamma (CST Cat#2619S, RRID: AB_2070984, 1:200), mouse Anti-Flag (Sigma Cat#F1804, RRID: AB_262044, 1:1000) and mouse Anti-β-actin (Sigma Cat#A5316, RRID: AB_476743, 1:2000), were incubated respectively overnight at 4 °C. After washing with 0.1% TBST, the horseradish peroxidase (HRP)-conjugated anti-rabbit or anti-mouse IgG were incubated at room temperature for 1 h (ZSGB-BIO Cat#ZB-2305, RRID: AB_2747415) and the bands were detected with ECL reagent (Thermo Scientific, 34580).

For immunoprecipitation, HA-tagged HP1γ and Flag-tagged RBPJ were co-transfected into HEK293T cells. After 24 h of transfection, the cells were cross-linked with 37% formaldehyde and terminated cross-linking with 2.5 M glycine. Then the cells were collected in lysis buffer (50 mm Tris–HCl, pH = 7.6, pH = 8.0, 5 M NaCl, 0.5% Nonidet P-40) with protease inhibitors and the ultrasonic was used to lyse the cells. Protein G-agarose beads and antibodies were added to cell lysates, followed by overnight incubation at 4 °C with end-over-end rotation. Cell lysates and immunoprecipitates were analyzed by Western blot for the indicated proteins.

### Immunofluorescence

The experiments were performed following standard procedures. Rabbit Anti-HP1gamma (CST Cat#2619S, RRID: AB_2070984, 1:200) and mouse anti-RBPJ (Santa Cruz Cat#sc-271128, RRID: AB_10610612, 1:200) were used to detect endogenous proteins. DAPI (Sigma, D8417) was used to mark the nucleus. Alexa Fluor 488 conjugated anti-rabbit (CST Cat#4412, RRID: AB_1904025, 1:200) and Alexa Fluor 546 conjugated anti-mouse (Thermo Cat#A11030, RRID: AB_2737024, 1:200) secondary antibodies were used to achieve visualization of protein localization. Images were captured using a Zeiss LSM 880 confocal laser scanning microscope (Zeiss, Germany) with a 63 × oil objective lens.

### Quantitative RT-qPCR

Using the RNeasy Mini Kit (QIAGEN, Cat. No. 74104), we isolated total RNA from all experimental specimens, followed by reverse transcription with the GoldScript cDNA Synthesis Kit (Invitrogen c81401190) for cDNA preparation. qRT-PCR was performed using the Talent qPCR PreMix (SYBR Green) kit (TIANGEN Biotech, Beijing, China) in LightCycler 480 (Roche). The primers used are shown below.

β-actin:5’-GTGTGTGACAATGGCTCTGG-3’, 3’-TCCCAGTTGGTGATGATGCC-5’.

Hes1:5’-CCTGTCATCCCCGTCTACAC-3’, 3’-CACATGGAGTCCGCCGTAA-5’.

Hes5:5’-AGAAAAACCGACTGCGGAAG-3’, 3’-GCTTTGCTGTGCTTCAGGTA-5’.

Hey1:5’-GTTCGGCTCTAGGTTCCATGT-3’,3’-CGTCGGCGCTTCTCAATTATTC-5’ Hes4:5’-CAGAAAAGAGAGCTCCCGC-3’, 3’-GGTACTTGCCCAGAACGG-5’.

p21: 5’-TGTCCGTCAGAACCCATGC-3’, 3’-AAAGTCGAAGTTCCATCGCTC-5’.

β-actin was used to normalize the relative transcript level of each gene. The results were calculated according to the 2^−ΔΔCt^ method.

### Tumorigenesis assay

Six-week-old female Balb/c nude mice, obtained from the Laboratory Animal Resources Center of Tsinghua University, were used for the tumorigenesis study. Briefly, 1 × 10⁶ LoVo cells of different stable transgenic lines were subcutaneously inoculated into the right flank of each mouse. Tumor growth was monitored by measuring the major (a) and minor (b) axes of the tumor with vernier calipers at regular intervals, and tumor volume was computed using V = 1/2 × a × b^2^. Upon completion of the experiment, mice were euthanized humanely, and xenograft tumors were excised and weighed for subsequent analysis.

### High-risk gene detection

CIPHER, a genome-wide gene-phenotype relationship prediction method based on the disease phenotype and OMIM Database (https://omim.org), was used to detect high-risk genes of CRC. Datasets MIM 266600, MIM 114500, MIM 137280 and MIM 613659 were used to perform CIPHER.

Genes in the “Chromosome and associated proteins” sets were directed from KEGG. The directed gene set was intersected with the gene set from CIPHER and 64 genes were selected for further survival testing.

### Statistical analysis

We performed survival analysis in GEPIA (RRID: SCR_018294, http://gepia.cancer-pku.cn/),. Survival data of patients were from the TCGA COAD dataset (https://portal.gdc.cancer.gov/), and disease-free survival time was used to for the Log-rank test.

Pan-cancer expression analysis of CBX3 was performed using the TCGA pan-cancer dataset via GEPIA. For differential expression analysis, patients from the TCGA COAD dataset were ordered by CBX3 expression level; the top 25% were defined as the high expression group, and the bottom 25% were defined as the low expression group. Differential expression analysis was performed using the DESeq2 package of R, and genes with adjusted P-value < 0.05 and |log2 fold change|> 1.3 were deemed as significantly differentially expressed.

GO and KEGG enrichment analyses (RRID: SCR_012773) were performed using the clusterProfiler package of R (RRID: SCR_016844) with the significant differentially expressed genes as input.

Pearson correlation coefficients between CBX3, BRAF, NRAS, KRAS, EGFR, and genes in the Notch signaling pathway were calculated based on the TCGA COAD expression profile, with P-values of correlation analysis visualized in a heatmap.

GraphPad Prism (RRID: SCR_002798) and Excel (RRID: SCR_016137) were used to calculate the mean and standard deviation (SD) for all experimental data, as well as corresponding statistical analysis. The exact sample size and detailed statistical method for each assay are specified in the corresponding figure legend.

## Results

### HP1γ is the highest-risk epigenetic factor in CRC

**Fig. 1:**
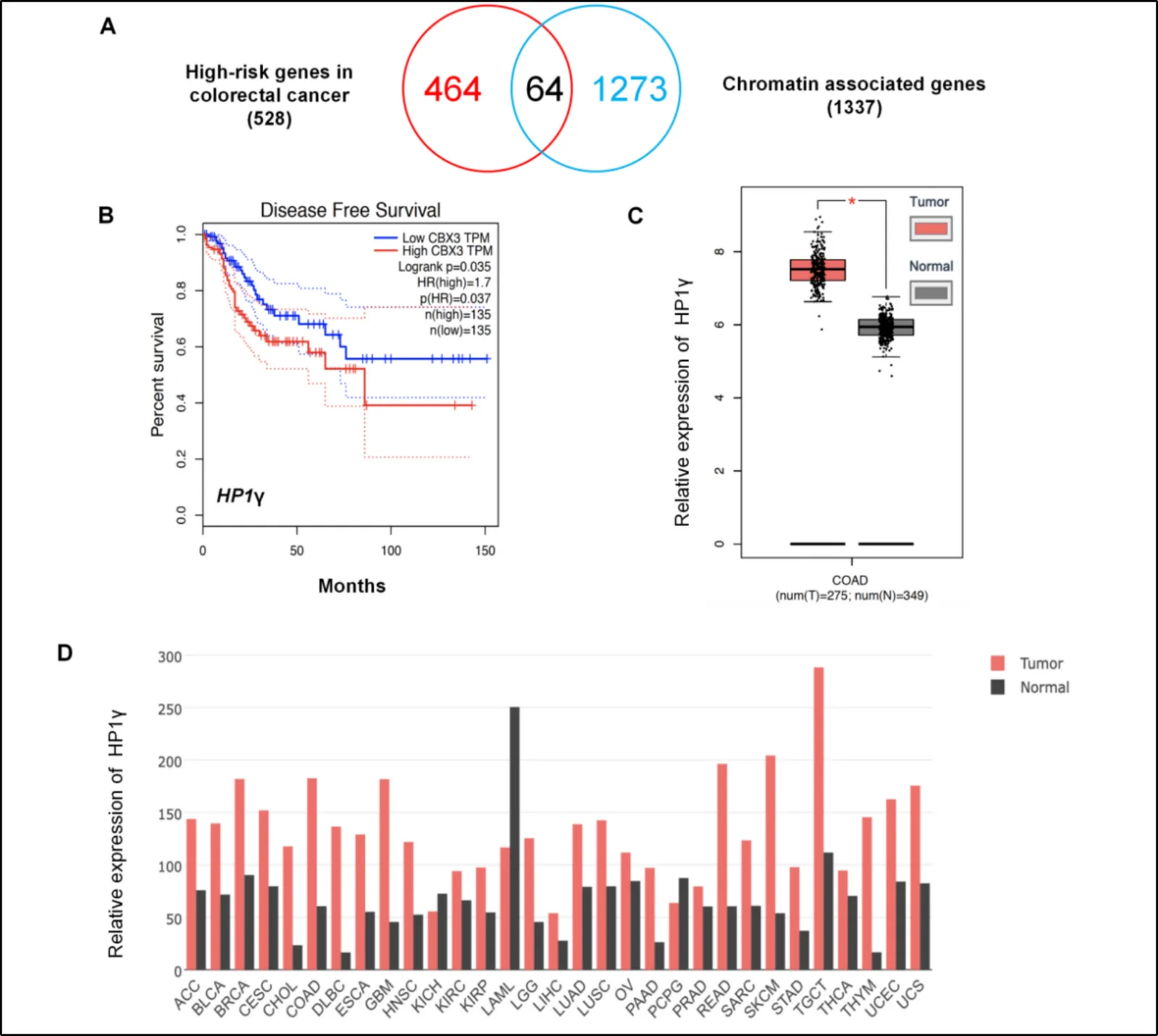
HP1γ is the highest-risk epigenetic factor in CRC **(A)** CIPHER (network-based genome-wide prediction of disease genes) was used to discover 528 high-risk genes. 64 high-risk genes related to epigenetic regulation were selected via intersecting with the “chromosome and associated proteins” gene set from KEGG. (**B**) Disease-free survival time was calculated using the public resource GEPIA. Based on the median expression level of HP1**γ** (standardized to TPM, total n = 270), enrolled patients were split into two groups for survival analysis. Log-rank test was performed to calculate the p-values for survival comparison, with the corresponding p-values annotated in the figures. The 95% confidence intervals of the survival curves are indicated by dotted lines. **(C)** Box plot showing relative expression of HP1γ in COAD tumor (n = 275) and adjacent normal (n = 349) tissues. P-value was determined by one-way ANOVA. (**D**) The gene expression profile across all tumor samples and matched paired normal tissues was retrieved from the GEPIA database. Expression of HP1γ was significantly elevated in almost all types of cancer except for KICH, LAML, and PCPG. Data source: TCGA COAD dataset via GEPIA (http://gepia.cancer-pku.cn/)

To identify essential genes associated with human CRC, we first performed a network-based and genome-wide prediction and found 528 high-risk genes. By intersecting those high-risk genes with the KEGG gene set “chromosome and associated protein”, 64 genes were shown in both lists (Fig. 1A). To explore the effect that they have on clinical outcomes, we analyzed the DFS rate of the 64 genes, respectively, by Gene Expression Profiling Interactive Analysis (GEPIA). There are three genes whose expression level had a significant impact on survival time, including *CBX3*, *RCC1*, and *TAF1* (Fig. 1B, S1A). Specifically, *CBX3* encodes HP1γ, an epigenetic regulator in the HP1 family, while *RCC1* regulates chromatin condensation and *TAF1* encodes a subunit of the transcription initiation factor TFIID. COAD is one of the most dominant pathological types of CRC. According to GEPIA analysis, a high level of RCC1 is related to COAD, which is not in line with the data from DFS, whereas TAF1 has no significant change between normal and COAD (Figure S1A, B). Therefore, we focused on HP1γ, which is highly expressed in COAD, and its elevation significantly reduces the DFS rate (Fig. 1B, C).

Interestingly, another two HP1 family members, HP1α and HP1β, have no statistically significant impact on DFS (Figure S1C), suggesting the specific role of HP1γ in DFS related to CRC progression. Most interestingly, the expression level of HP1γ is largely upregulated in the majority of cancers except for KICH (Kidney Chromophobe), LAML (Acute Myeloid Leukemia) and PCPG (Pheochromocytoma and Paraganglioma) (Fig. 1D), indicating HP1γ could serve as a hall marker for cancer diagnosis, and HP1γ is the potential key epigenetic factor which participates in CRC progression.

### HP1γ is elevated in COAD

**Fig. 2:**
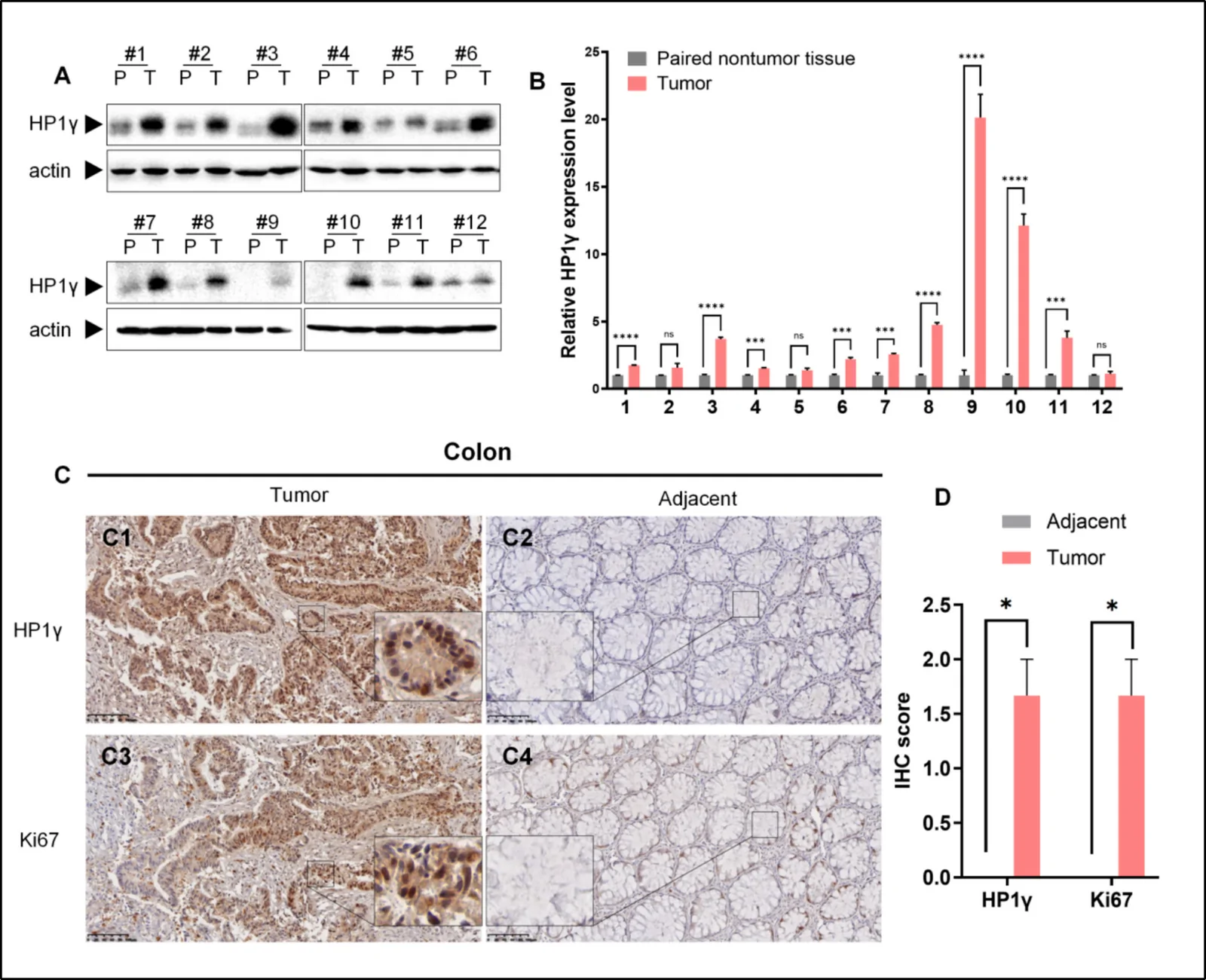
HP1γ is elevated in COAD **(A)** Representative Western blot images showing HP1γ protein levels, with β-actin for loading normalization. **(B)** Normalized HP1γ protein expression relative to β-actin. Data are shown as mean ± SD. *n* = 3. ****p < 0.0001(paired Student’s t-test). (**C**) Immunohistochemical staining is shown for HP1γ and Ki67 in human COAD tissues, including tumor tissues (D1, D3) and adjacent non-tumor tissues (D2, D4). Scale bars, 100 µm. (**D**) Quantification of IHC staining intensity (H-score) for HP1γ and Ki67 in COAD tumor and adjacent non-tumor tissues. Staining intensity was scored from 0 to 3, positive cell proportion was scored from 0 to 3, and the final IRS score was calculated as the product of intensity and proportion scores, with statistical significance determined by paired Student’s t-test. Data are presented as mean ± SD. *n* = 5. *p < 0.05

Based on the above bioinformatic analysis, HP1γ is highly expressed in patients with COAD compared to healthy donors. To confirm the elevation of HP1γ in COAD, we first performed a Western blot with samples from the tumor and adjacent non-tumor tissues as controls in twelve patients. The patients were of different genders, clinical stages, and degrees of tumor differentiation (Table 1). Consistent with bioinformatic analysis (Fig. 1C), with a specific antibody to recognize HP1γ, we found that the expression level of HP1γ is dramatically increased in tumor tissues compared with controls from almost all these patients (Fig. 2A), and the quantitative analysis confirmed the statistical significance (Fig. 2B).

In addition, with the immunohistochemical experiment, in contrast to tumor-adjacent tissue, the tumor tissue has an irregular cell shape and more enrichment of Ki67 (Fig. 2C, D). Consistent with the results of the western blot, the intensity of HP1γ was also elevated (Fig. 2C, D) in tumor tissue. Taken together, both western blot and immunochemical assays confirmed the conclusion that HP1γ is highly expressed in COAD.

### HP1γ exerts a dose-dependent oncogenic role in COAD cells *in vitro*

**Fig. 3:**
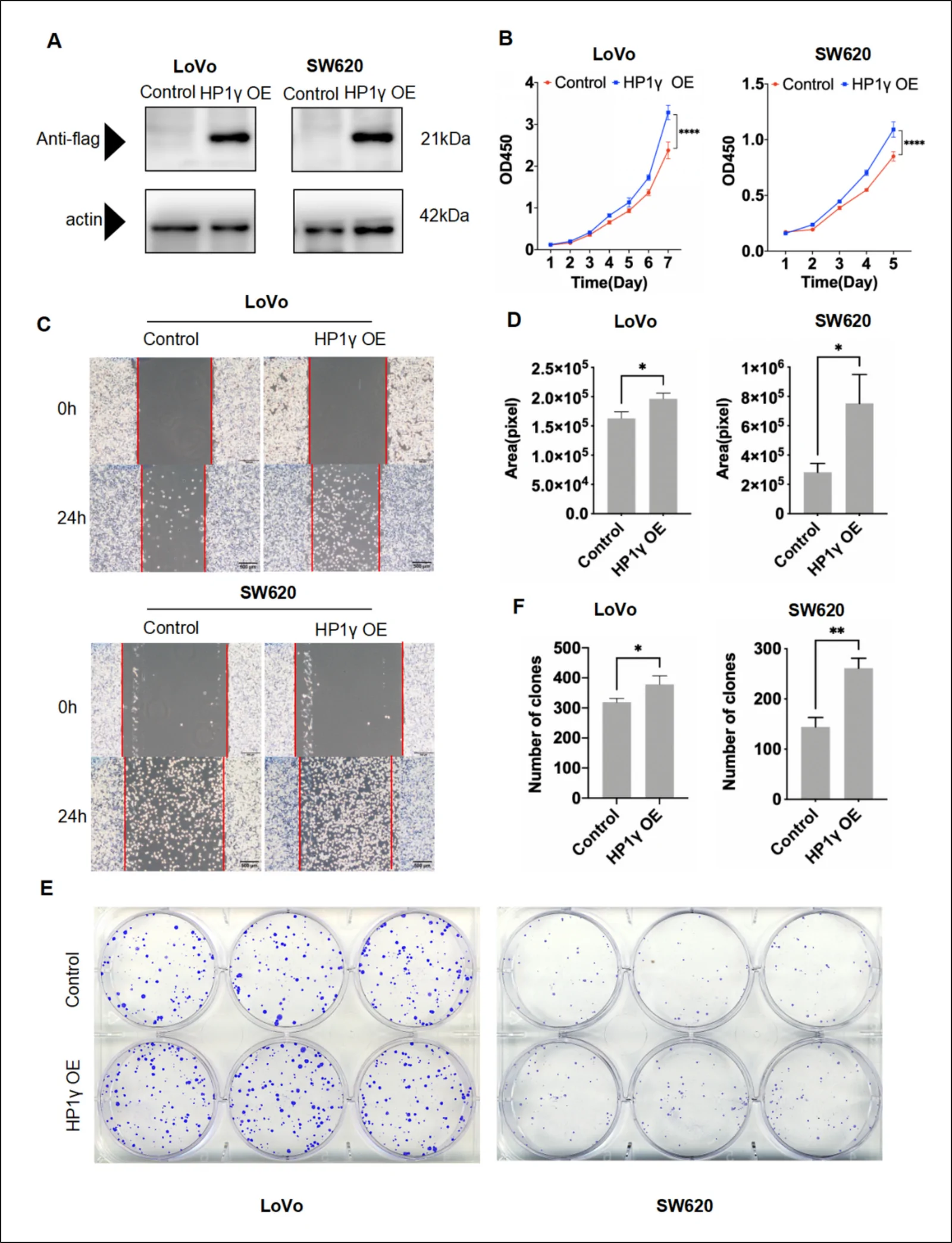
HP1γ promotes tumor cell growth *in vitro* (**A**) Western blot assays were used to characterize the expression of HP1γ in LoVo and SW620 stable cell lines. An empty vector was transfected into LoVo or SW620 cells as controls. (**B**) The proliferation of LoVo-control cells (red line) and LoVo-HP1γ overexpression cells (blue line) was determined by Cell Counting Kit-8 (CCK-8) shown on the left. The proliferation of SW620-control cells (red line) and SW620-HP1γ overexpression cells (blue line) was determined by CCK-8 shown on the right. *n* = 3, mean ± SD, ****P < 0.0001. (**C**) Cell migration assay. Indicated stable cell lines were scratched with sterile pipette tips. The pictures were captured by microscopy after 0 h and 24 h, respectively. Scale bars, 500 µm. (**D**) The statistical results of the scratch area were measured by the software ImageJ. *n* = 3, mean ± SD, *P < 0.05. (**E**) These photomicrographs show colony formation assays of the indicated stable cell lines. (**F**) ImageJ was utilized to enumerate the number of formed clones. *n* = 3, mean ± SD, *P < 0.05; **P < 0.01

The preferential elevation of HP1γ in COAD indicates it might be involved in tumor progression. To test this idea, we overexpressed HP1γ in both LoVo and SW620 cell lines (Fig. 3A), which are both derived from COAD patients. We first performed the CCK-8 (Cell Counting Kit-8) test. In contrast to the control, overexpression of HP1γ significantly increases the number of cells, suggesting HP1γ promotes tumor cell proliferation (Fig. 3B). We then looked at the migration capability of the cells. The scratch test showed HP1γ overexpressing cells migrate faster than controls, and the statistical results confirmed the significance of the migration. (Fig. 3C,D). Last, we did a clonogenic assay. As we expected, the overexpression of HP1γ also increases the number of tumor cell colonies (Fig. 3E, F). To confirm the dose-dependent oncogenic role of HP1γ, we performed siRNA-mediated knockdown of HP1γ in SW620 cells (Figure S2A). CCK-8 assays showed that HP1γ knockdown significantly suppressed cell proliferation (Figure S2B). Scratch migration assays and colony formation assays further confirmed that HP1γ knockdown reduced the migration capacity (Figure S2C, D) and clonogenic potential of SW620 cells (Figure S2E, F). Based on *in vitro* assays, HP1γ exerts a dose-dependent oncogenic role in COAD cells by promoting proliferation, migration and colony formation.

### HP1γ promotes tumor growth *in vivo*

**Fig. 4:**
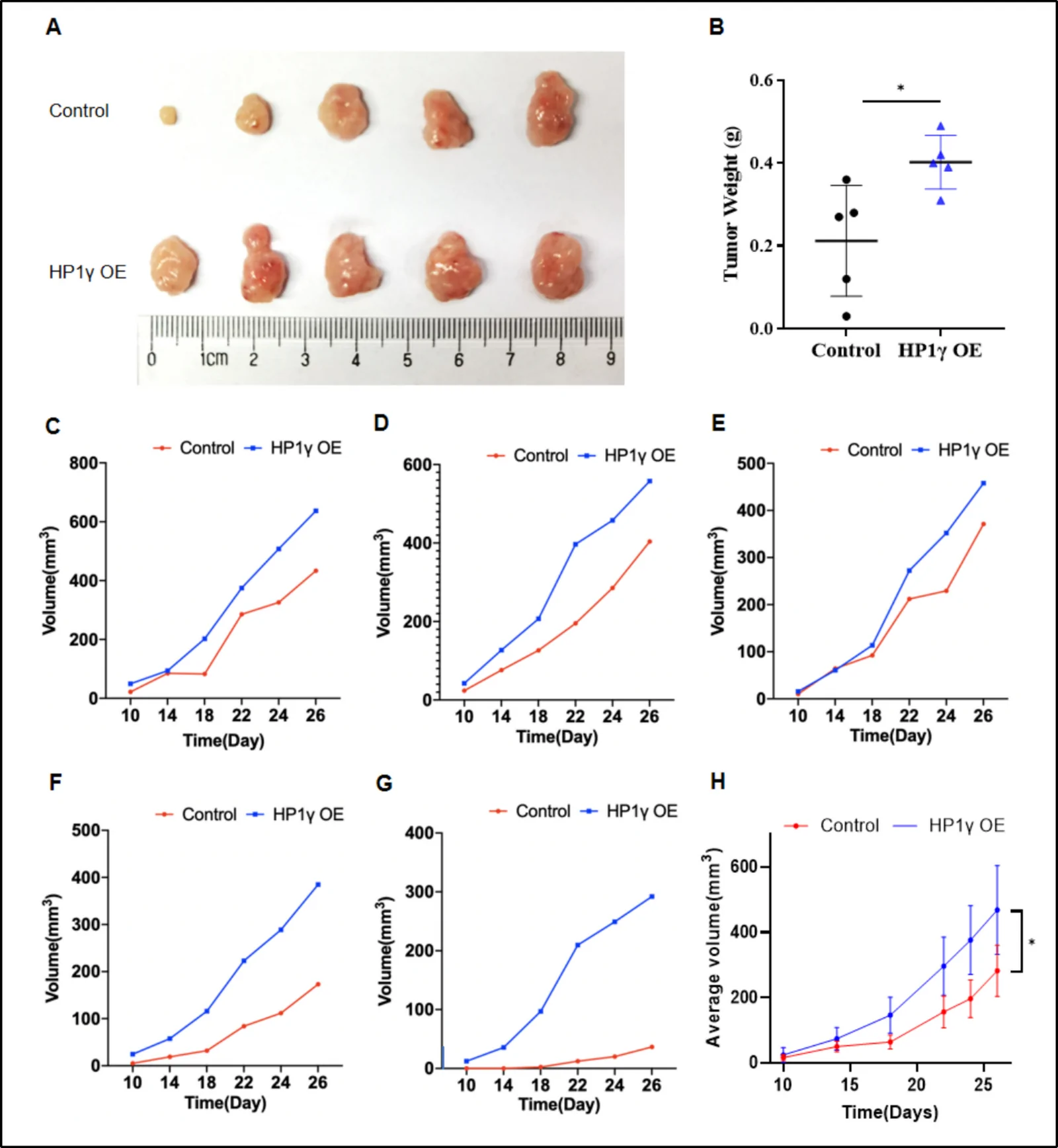
HP1γ promotes tumor growth *in vivo* For each indicated stable cell line, five nude mice received subcutaneous injections of 1 × 10^6^ cells per animal. (**A**) This figure shows the isolated tumors. (**B**) It is the statistical result of tumor weight. *n* = 5, mean ± SD, *P < 0.05. (**C-G**) Tumor growth curve of each nude mouse. The volume is calculated from the length and width measured by vernier calipers, and the day of injection is day 0. (**H**) The mean tumor volume change curve of each group of nude mice, by one-tailed Student’s t-test at the final endpoint. *n* = 5, mean ± SD, *P < 0.05

Given that the overexpression of HP1γ promotes tumor cell proliferation, migration, and colony formation *in vitro*, we performed a tumorigenesis experiment by injecting LoVo cell lines, in which HP1γ is stably overexpressed, into nude mice. Compared with the control, the elevation of the HP1γ increases tumor size at different time points, and the statistical results showed the significance of the enlargement (Fig. 4A). Another independent experiment also gave a similar result: HP1γ promotes tumor growth (Figure S3A). After three weeks, we dissected the tumor from the mice, in addition to size (Fig. 4C-H), the tumor weight significantly increased under upregulated HP1γ (Fig. 4B, Figure S2B). Taken together, the tumorigenesis experiments further support that HP1γ intrinsically promotes tumor progression and confirm our bioinformatic analysis that HP1γ is a high-risk epigenetic factor in COAD.

### HP1γ suppresses the Notch signaling pathway

The progression of COAD is often related to aberrant cell signaling pathways, including the well-known WNT, EGFR, MAPK/PI3K TGF-β, and Notch signaling pathways. The EGFR and MAPK/PI3K signaling normally function by modulating downstream pathways such as JNK and NF-κB pathways. To determine which signaling pathway is coordinated by transcriptional repressor HP1γ, we looked at the changes of the downstream key transcription factor for each signaling pathway by GEPIA. As we can see, *TCF* for WNT, *C-Jun* for JNK, *SMAD1* for TGF-β, *RelA* for NF-κB, and *TP53* for apoptosis, either no change or an increase, which is unlikely to be directly modulated by the upregulation of the transcriptional repressor HP1γ (Figure S4).

**Fig. 5:**
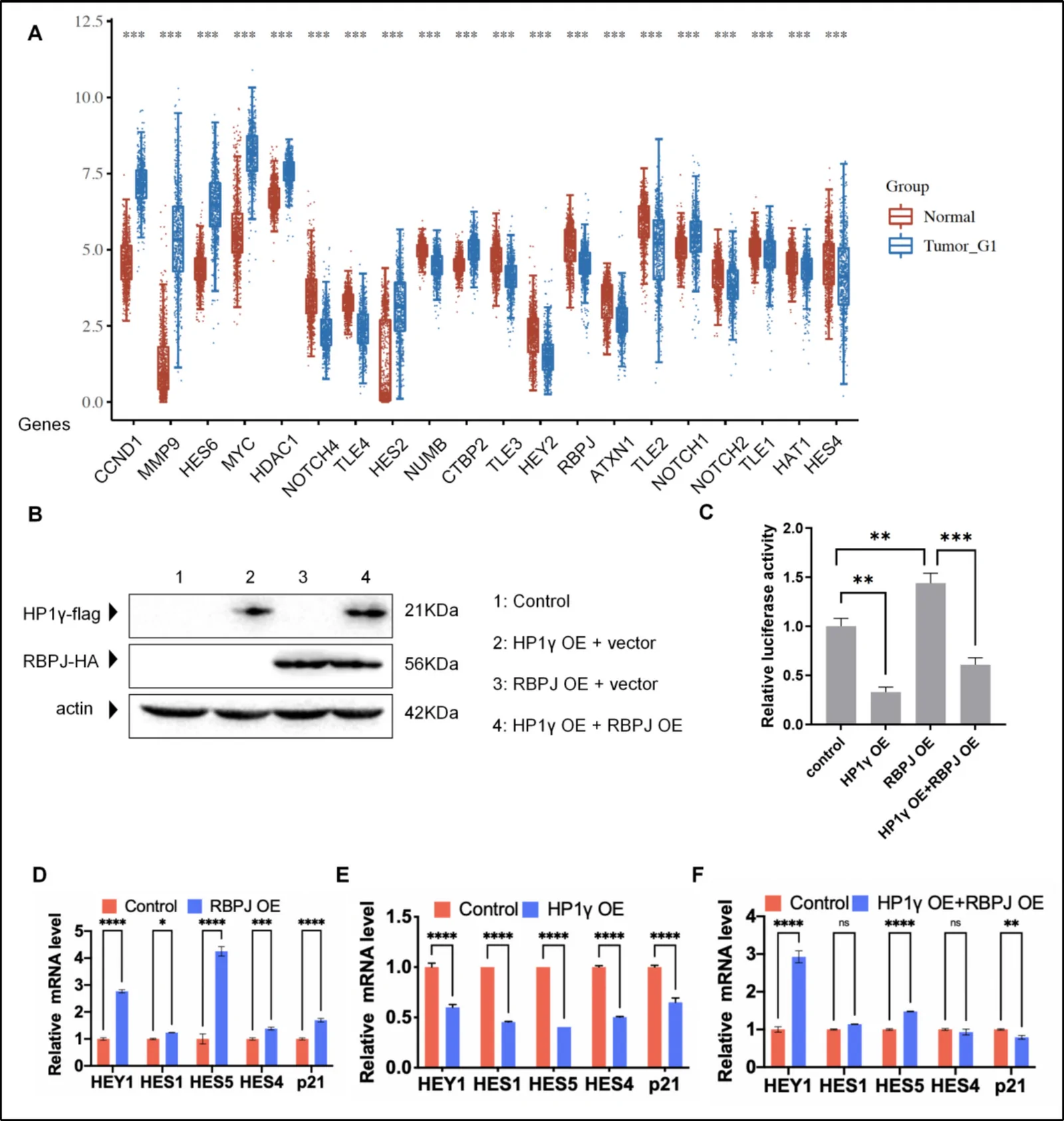
HP1γ suppresses the Notch signaling pathway (**A**) The expression distribution of genes in Notch signaling in colon tumor tissues (blue) and normal tissues (red) at the mRNA level. The number of tumor samples is 275, and the number of normal samples is 349. The significance of the two groups of samples was tested by Wilcox. ***P < 0.001. (**B**) Western blot analysis of LoVo stable cell lines. Two empty vectors were transfected into LoVo cells as a control. Flag-tagged HP1γ and an empty vector were transfected into LoVo cells (HP1γ OE + vector) as the stable cell line of HP1γ overexpression. HA-tagged RBPJ and an empty vector were transfected into LoVo cells (RBPJ OE + vector) as the stable cell line of RBPJ overexpression. Flag-tagged HP1γ and HA-tagged RBPJ were transfected into LoVo cells (HP1γ OE + RBPJ OE) as the rescue stable cell line. (**C**) The Notch reporter system for the correlation check. After the overexpression of HP1γ, the intensity of luciferase significantly decreased, while with the overexpression of RBPJ, the intensity of luciferase significantly increased; moreover, the overexpression of HP1γ together with RBPJ increased the intensity of luciferase compared to the individual overexpression of HP1γ group. (**D-F**) The qPCR analysis of typical Notch target genes (*HEY1*, *HES1*, *HES5*, *HES4*, *p21*) in the indicated stable cell lines. (B-D) *n* = 3, mean ± SD, ns, no significance; *P < 0.05; **P < 0.01; ***P < 0.001; ****P < 0.0001

Interestingly, RBPJ, which is the key transcription factor of Notch, is significantly reduced in the cancer tissue from COAD (Figure S4), suggesting a potential link between HP1γ and the Notch signaling pathway component RBPJ. To determine the relationship between HP1γ and Notch target genes, we look at the transcriptional level of HP1γ and the Notch target genes from the tumor tissues of COAD patients. The transcription of the majority of Notch target genes was significantly reduced (Fig. 5A). To confirm our conclusion that HP1γ prevents Notch signaling, we performed the Notch signaling activity assay. The mammalian Notch-luciferase reporter coupled with the vector carrying GFP, HP1γ, RBPJ, HP1γ plus RBPJ respectively were transfected into HEK293T cells, the cells were collected and lysed after 1-day transfection, and the overexpression was confirmed by western blot (Fig. 5B), and then the activity of the luciferase was measured.

In contrast to control expressing GFP, the overexpression of HP1γ significantly reduces the activity of luciferase (Fig. 5C), supporting the repressor role of HP1γ in Notch signaling. Consistent with the previous report, the upregulated RBPJ is positively correlated with luciferase activity. Most interestingly, the increase of the luciferase activity through overexpressing RBPJ can be suppressed by the co-expression of HP1γ, further confirming that HP1γ preferentially downregulates Notch signaling (Fig. 5C).

To demonstrate the suppressor role of HP1γ in Notch signaling, we detected the transcription level of the endogenous Notch-targeting genes under overexpressing of HP1γ in LoVo cells. The coding sequence of GFP, HP1γ, RBPJ, and HP1γ plus RBPJ was integrated into the lentiviral vector, and then the generated LoVo stable cell lines were used for qRT-PCR (quantitative real-time PCR) analysis. As a positive control, increasing RBPJ can successfully upregulate Notch target genes, including *HEY1*, *HES1*, *HES5*, *HES4*, and *p21*(Fig. 5D), while HP1γ significantly reduced the transcripts of the same set of genes (Fig. 5E).

In line with the luciferase assay, HP1γ also prevents the transcriptional activity triggered by RBPJ except for *HEY1* and *HES5* (Fig. 5F). Altogether, in addition to GEPIA prediction, the Notch reporter system, as well as the targeted endogenous genes assays, support that HP1γ specifically regulates the Notch signaling pathway.

### HP1γ is associated with RBPJ

**Fig. 6:**
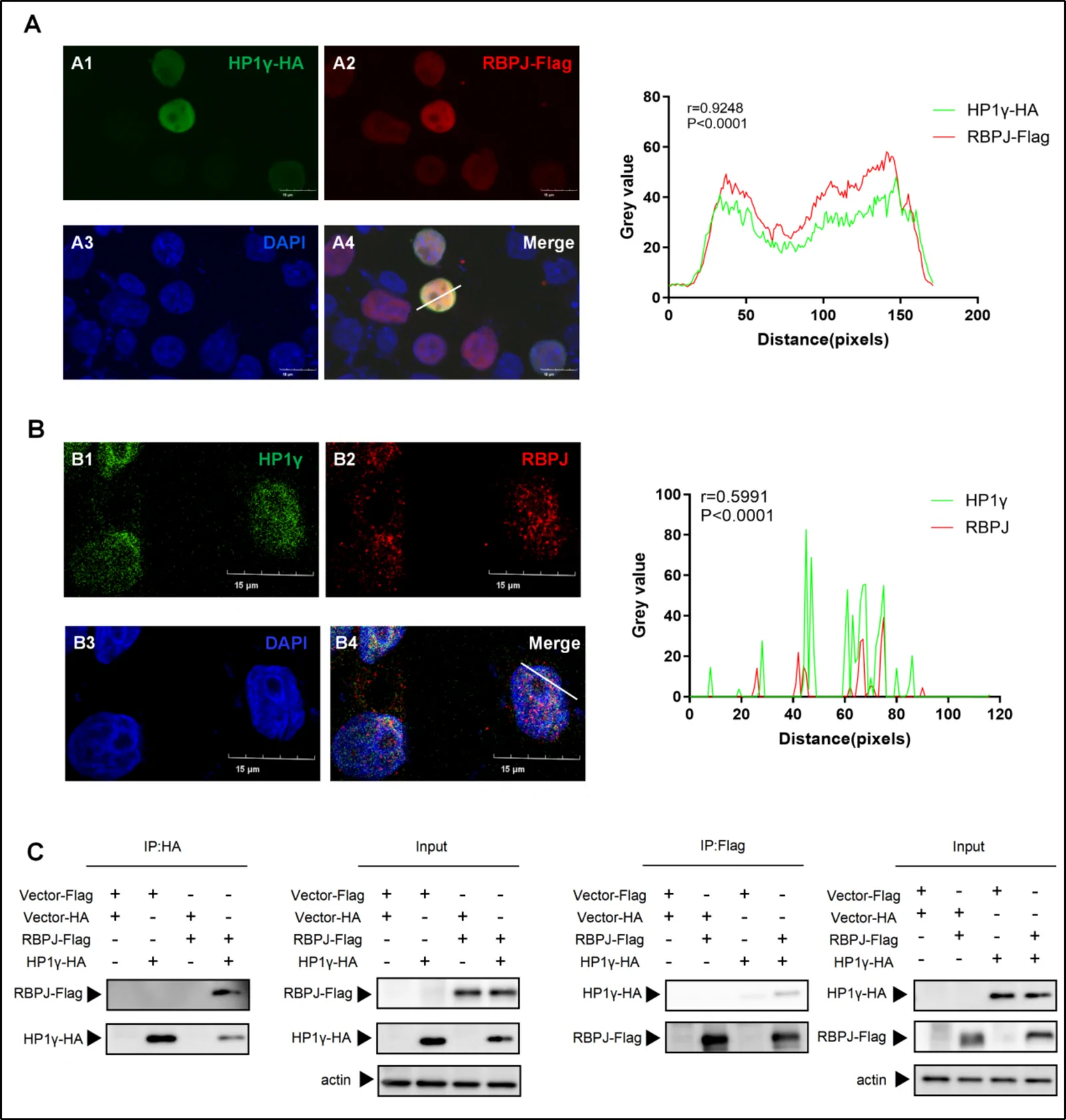
HP1γ interacts with RBPJ (**A, B**) Co-localization of HP1γ and RBPJ in the nucleus of LoVo cells. (A1-A4) Cells co-expressing HA-HP1γ and Flag-RBPJ were stained with anti-HA (green) and anti-Flag (red) antibodies. (B1-B4) Endogenous HP1γ (green) and RBPJ (red) were detected with specific antibodies. DAPI (blue) stains the nucleus. Merged images (A4, B4) show overlapping signals. The right panels present the fluorescence intensity profile along the white line in the merged image, with the Pearson correlation coefficient for co-localization indicated which is performed by Image J. Scale bars, 15 µm. (**C**) HP1γ interacts with RBPJ. HA-tagged HP1γ (HP1γ-HA) and Flag-tagged RBPJ (RBPJ-Flag) were co-expressed in HEK293T cells. HA and Flag-tagged vectors (Vector-HA and Vector-Flag) were used as controls

Since HP1γ is a well-known transcription repressor, HP1γ may suppress Notch signaling at the transcription level by repressing the transcription activator RBPJ. So, we first overexpressed the tagged HP1γ and RBPJ in HEK293T cells, and then did an immunofluorescence assay; both HP1γ and RBPJ were in the cell nucleus, marked by DAPI and largely colocalized with each other, indicating they may interact with each other (Fig. 6A). To confirm our observation, antibodies against endogenous HP1γ and RBPJ were used to perform cytological analysis. Consistent with the tagged proteins, the pattern of HP1γ is largely merged with that of RBPJ, indicating HP1γ might prevent the transcription of Notch target genes through RBPJ at the protein level, and HP1γ might directly interact with RBPJ (Fig. 6B).

To validate the interaction within them, we transiently overexpressed HA-HP1γ and Flag-RBPJ in HEK293T and performed the co-immunoprecipitation (Co-IP) experiment. Both HA-HP1γ and Flag-RBPJ can successfully recover each other in the precipitation detected by Western blot, supporting HP1γ physically interacting with RBPJ to regulate the transcription of Notch target genes (Fig. 6C). To map the interaction domains, we generated truncated constructs of HP1γ (CSD domain) and RBPJ (BTD domain). Co-IP assays demonstrated that the CSD domain of HP1γ specifically interacted with the BTD domain of RBPJ (Figure S5A). These results establish that the CSD-BTD interaction is necessary for HP1γ–RBPJ complex formation.

To determine the functional outcome of this interaction, we conducted chromatin immunoprecipitation followed with quantitative PCR (ChIP-qPCR). HP1γ and RBPJ were significantly co-enriched at the promoter regions of canonical Notch target genes (*HES1*, *HEY1*, *HES4*, *p21)* in LoVo cells, whereas no enrichment was detected at the negative control GAPDH promoter (Figure S5B).

### The rescue experiments further support the interaction between HP1γ and RBPJ

**Fig. 7:**
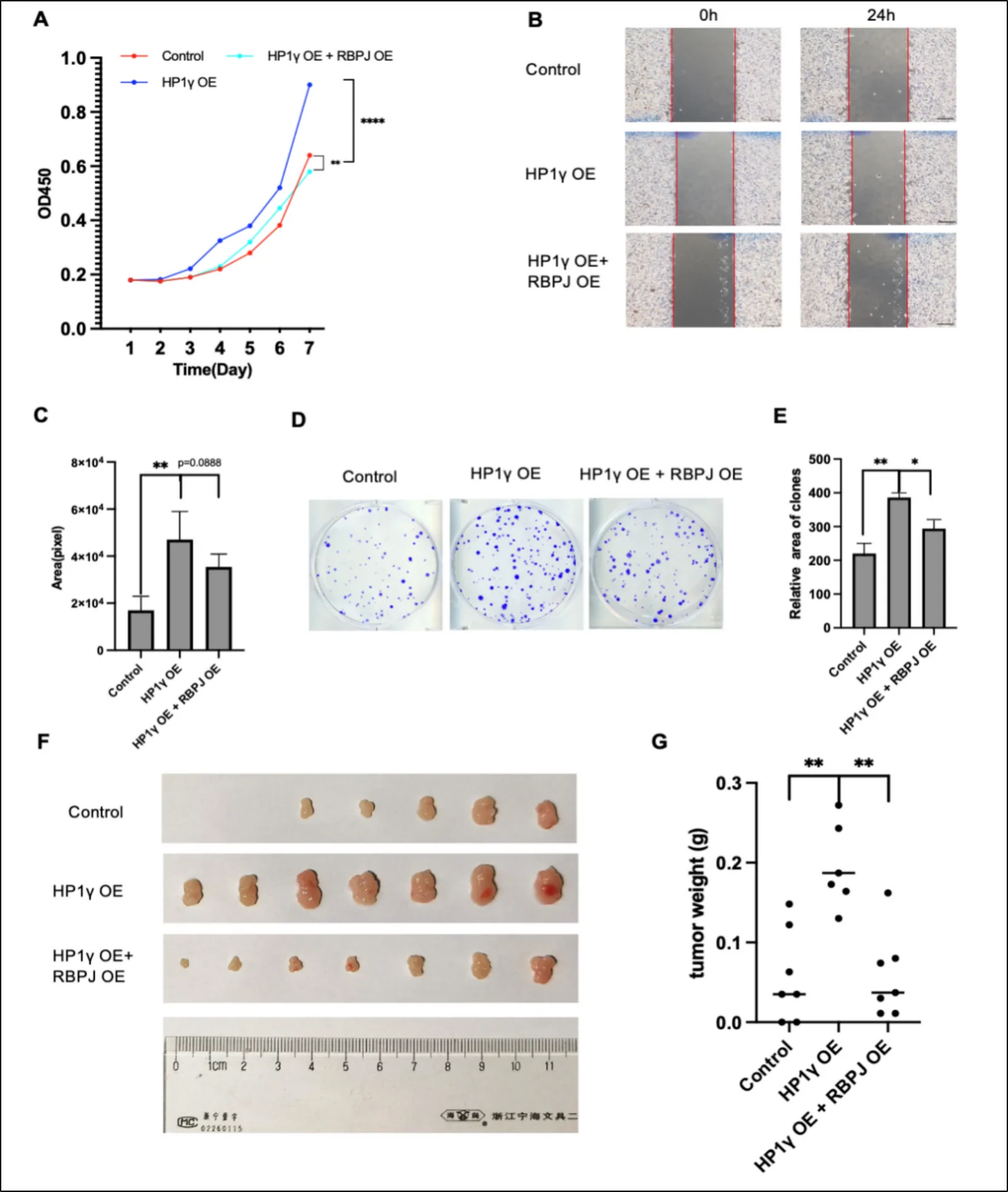
The rescue experiments for the tumor progression triggered by the HP1γ (**A**) The growth curves for LoVo-Control cells (red line), LoVo-HP1γ OE cells (blue line), and LoVo-HP1γ OE + RBPJ OE cells (cyan line). *n* = 3, mean ± SD, **P < 0.01; ***P < 0.001; ****P < 0.0001. (**B**) The cell migration assay of the indicated stable cell lines. Scale bars, 500 µm. (**C**) The migration area is measured by the software ImageJ. *n* = 3, mean ± SD, **P < 0.01. (**D**) The colony formation assay of the indicated stable cell lines. (**E**) The clone numbers were counted by ImageJ. *n* = 3, mean ± SD, *P < 0.05; **P < 0.01. (**F**) Tumor weight of different nude mice groups in (**G**). *n* = 7, mean ± SD, ns, no significance; **P < 0.01

To verify that HP1γ suppresses the Notch target genes by interaction with RBPJ and then promotes COAD progression, we first performed the CCK-8 test, cell scratch test, and clonogenic assay on the HP1γ overexpression cell line and the rescued cell line, which simultaneously expresses RBPJ and HP1γ. The rescued cell line showed a weaker ability for proliferation, migration, and clone formation, which tallied our expectations (Fig. 7A-E). We inferred that HP1γ might act as a repressor that prevents the activity of RBPJ.

Next, we tried to rescue the rapid tumor growth triggered by HP1γ overexpression *in vivo*. RBPJ was overexpressed together with HP1γ in LoVo cells and the injection was performed (Figure S4A). After weighing the tumors for 25 days after injection, we found that simultaneously overexpressing RBPJ and HP1γ recovered tumors to a similar size as the control (Fig. 7F, G). This interesting phenomenon further confirms our conjecture that HP1γ acts as a repressor, which retains the transcriptional activity of RBPJ and promotes tumor growth. The increase of RBPJ will release the RBPJ from HP1γ control, thus preventing tumor growth.

## Discussion

Colorectal cancer (CRC) is one of the most prevalent and lethal malignancies worldwide, while the epigenetic regulatory mechanisms underlying its progression remain largely elusive. In this study, we identified the epigenetic regulator HP1γ as a critical driver of CRC progression, which is distinct from the other two HP1 family members (HP1α and HP1β) due to its euchromatin localization and direct transcriptional regulatory function. Through genome-wide prediction and disease-free survival (DFS) correlation analysis, we found that high HP1γ expression is significantly associated with poor DFS in CRC patients, and we further confirmed the upregulation of HP1γ in clinical samples of colon adenocarcinoma (COAD), the major histological subtype of CRC. Functional experiments demonstrated that HP1γ exerts a dose-dependent oncogenic effect in CRC. Overexpression of HP1γ significantly enhanced the proliferation, migration and colony formation of CRC cells *in vitro*, while HP1γ knockdown markedly inhibited these malignant phenotypes, which was further validated by *in vivo* xenograft models showing that HP1γ overexpression significantly promoted tumor growth and increased tumor volume and weight. These findings establish HP1γ as a functional oncogenic epigenetic regulator in CRC, filling the gap in the understanding of HP1γ’s role in CRC tumorigenesis. The evolutionarily conserved Notch signaling pathway plays a complex, context-dependent role in CRC, with previous studies reporting both tumor-promoting and tumor-suppressive effects of different Notch receptors in CRC. For instance, aberrant activation of Notch1 has been shown to drive CRC initiation, while Notch2 expression is negatively correlated with poor prognosis in CRC patients, indicating the highly complicated regulatory network of Notch signaling in CRC. Notably, the epigenetic regulatory mechanisms of Notch signaling in CRC remain poorly characterized, which limits our understanding of its context-dependent function. Strikingly, our study revealed that HP1γ promotes CRC progression by directly repressing the Notch signaling pathway. Bioinformatic analysis showed that the expression of canonical Notch target genes is significantly downregulated in COAD patients with high HP1γ expression, indicating the inactivation of Notch signaling in HP1γ-high tumors. We further validated this regulatory effect *in vitro*. Overexpression of HP1γ significantly reduced the transcription of Notch target genes in CRC cells, and the inhibition of Notch signaling activity was confirmed by Notch luciferase reporter assay. As a well-characterized transcriptional repressor, HP1γ could modulate Notch signaling through two potential mechanisms. Direct transcriptional silencing of Notch-related genes via targeting their regulatory sequences, or indirect repression via interacting with core components of the Notch pathway. Here, we demonstrated that HP1γ directly physically interacts with RBPJ, the core nuclear transcriptional factor of the canonical Notch pathway. This finding is consistent with a previous study in *Drosophila* showing that HP1c (the mammalian HP1γ homolog) interacts with Su(H) (the mammalian RBPJ homolog) to regulate Notch signaling in intestinal stem cells, suggesting the evolutionarily conserved function of HP1 family proteins in Notch pathway regulation. We further confirmed this interaction via co-localization analysis, co-immunoprecipitation assays, and both *in vitro* and *in vivo* rescue experiments. Mechanistically, we found that HP1γ is recruited to the promoters of Notch target genes via its direct interaction with RBPJ, thereby reinforcing a transcriptionally repressive state and leading to comprehensive inactivation of the Notch pathway, which ultimately drives CRC progression. This model is further supported by our ChIP-qPCR results showing that the HP1γ-RBPJ complex is significantly co-enriched at the promoters of canonical Notch target genes. Given the canonical function of HP1γ in recognizing the repressive histone mark H3K9me3 and mediating chromatin compaction, we speculate that the recruitment of HP1γ may further stabilize the repressive chromatin state at Notch target gene loci, which constitutes an auxiliary epigenetic regulatory mechanism. As far as we are aware, this study provides the first evidence demonstrating that HP1γ directly interacts with RBPJ to repress Notch signaling and drive CRC progression, which provides novel insights into the epigenetic regulatory mechanism of the context-dependent Notch pathway in CRC. Our findings also highlight the clinical potential of HP1γ, whose aberrant upregulation in COAD tissues and significant correlation with poor patient prognosis indicate that HP1γ could serve as a promising prognostic biomarker for CRC, and targeting the HP1γ-RBPJ-Notch axis may provide a novel therapeutic strategy for CRC treatment.

There are several limitations of the current study that we should acknowledge herein. First, although we have verified the oncogenic role of HP1γ and its regulatory effect on the Notch pathway via *in vitro* cell experiments and xenograft models, we did not perform immunohistochemical staining for proliferation and apoptosis markers in tumor tissues, such as Ki67 and cleaved caspase-3, which limits our in situ mechanistic evidence at the histological level. Second, the physical interaction between HP1γ and RBPJ was mainly confirmed by exogenous overexpression systems. Further validation of this endogenous interaction under physiological conditions in CRC cells is still required. Third, the specific molecular mechanism underlying RBPJ downregulation in COAD tissues, including whether it is mediated by transcriptional repression or post-translational modification, has not been fully clarified in our study. Fourth, our ChIP-qPCR results support the co-occupancy of the HP1γ-RBPJ complex on Notch target gene promoters, but the auxiliary epigenetic mechanism mediated by HP1γ, such as H3K9me3-dependent chromatin condensation, needs further functional verification. Finally, our study mainly focused on the HP1γ-RBPJ-Notch axis, while the potential crosstalk between HP1γ and other CRC-related signaling pathways or epigenetic co-regulators was not explored in depth. The above limitations will be comprehensively addressed in our follow-up studies. We will further validate the endogenous HP1γ-RBPJ interaction and the H3K9me3-dependent chromatin regulatory mechanism, and explore the potential crosstalk between the HP1γ-Notch axis and other CRC-related signaling pathways. In addition, an intriguing question to be elucidated is whether HP1γ functions similarly in normal somatic tissues to regulate cell proliferation as in tumor cells. HP1γ is essential for heterochromatin formation, and chromatin dynamics are well-documented in tumor maintenance. It remains to be explored whether the heterochromatin state triggered by HP1γ overexpression is reversible, and whether reducing HP1γ expression to physiological levels can reverse the hyperproliferative phenotype of CRC cells. Furthermore, we will investigate the functional cooperation between HP1γ and KDM5A in the repression of Notch signaling, to fully delineate the oncogenic regulatory network of HP1γ in CRC.

## Supplementary Information

Below is the link to the electronic supplementary material.

Supplementary file1 (DOCX 2087 KB)

## Data Availability

The data that support the findings of this study are available from the corresponding author upon reasonable request.
