# Supplementary material for: HP1γ promotes the progression of colorectal cancer through interaction with RBPJ of the Notch signaling pathway
Source: Cell Biol Toxicol. 2026 Jul 1;42(1):108. doi: 10.1007/s10565-026-10205-z (PMC13593721; doi:10.1007/s10565-026-10205-z)
Supplement: Supplementary file 1 [file 10565_2026_10205_MOESM1_ESM.docx]

**Supplementary Figure S1**

**Figure S1. Clinical relevance of high-risk epigenetic genes and HP1 family members in CRC**

(A) Kaplan-Meier disease-free survival (DFS) analysis of COAD patients stratified by *RCC1* (left) and *TAF1* (right) expression (median cutoff, n=270 per gene). P-values were determined by log-rank test. Dotted lines represent the 95% confidence intervals of the survival curves. (B) Box plots showing relative mRNA expression of *RCC1* (left) and *TAF1* (right) in COAD tumor (n=275) and adjacent normal (n=349) tissues. P-values were determined by Wilcoxon rank-sum test.(C) Kaplan-Meier DFS analysis of COAD patients stratified by *CBX5* (HP1α, left) and *CBX1* (HP1β, right) expression (median cutoff, n=270 per gene). P-values were determined by log-rank test. Dotted lines represent the 95% confidence intervals of the survival curves. Data were obtained from the TCGA-COAD dataset via the GEPIA portal (http://gepia.cancer-pku.cn/)

**Supplementary Figure S2**

**Figure S2. HP1γ knockdown inhibits malignant phenotypes of SW620 cells *in vitro***

(A) RT-qPCR analysis of HP1γ protein expression in SW620 cells transfected with HP1γ-targeting siRNA or negative control siRNA (si-NC). GAPDH was used as a control.(B) CCK-8 proliferation assay of si-NC- and si-HP1γ-transfected SW620 cells. n=3, mean ± SD, ****P < 0.0001 (two-way ANOVA).(C) Representative images of scratch migration assays in si-NC- and si-HP1γ-transfected SW620 cells at 0 h and 24 h post-scratch. Scale bars: 500 μm.(D) Quantification of relative scratch closure area in (C) using ImageJ software. n=3, mean±SD, P < 0.01 (unpaired t-test).(E) Representative images of colony formation assays in si-NC- and si-HP1γ-transfected SW620 cells.(F) Quantification of colony number in (E) using ImageJ software. n=3, mean ± SD, P < 0.01 (unpaired t-test).

**Supplementary Figure S3**

**Figure S3. HP1γ promotes tumor formation *in vivo.***

(A) Independent parallel replicates of nude mice tumorigenic experiments. Fourteen nude mice were injected subcutaneously with 3 × 10^6^ cells/mouse for each of the indicated stable cell lines. The control group was established by infecting LoVo cells with the empty pCDH-EGFP lentiviral vector, so the control group is labelled “GFP OE”. The tumors were isolated after 3 weeks. (B) The tumor weight of different tumors in (A). n=14, mean ± SD, *, P<0.05.

**Supplementary Figure S4**

**Figure S4. Expression of key transcription factors in CRC-related signaling pathways in COAD**

The expression of downstream key transcription factors for each signaling pathway in colon tumor tissues (red) and normal tissues (gray). The number of tumor samples is 275, and the number of normal samples is 349. Data were obtained from the TCGA-COAD dataset via the GEPIA portal

**Supplementary Figure S5**

**Figure S5. HP1γ interacts with RBPJ BTD domain through the CSD domain and co-occupies Notch target gene promoters.**

(A) Co-immunoprecipitation (Co-IP) assays confirm that the chromoshadow domain (CSD) of HP1γ interacts with the beta-trefoil domain (BTD) of RBPJ. HEK293T cells were co-transfected with BTD-mCherry and CSD-eGFP constructs, followed by IP with anti-mCherry antibody. Input and IP samples were analyzed by Western blot.(B) Chromatin immunoprecipitation followed by quantitative PCR (ChIP-qPCR) assays show the enrichment of HP1γ (left) and RBPJ (right) at the promoters of canonical Notch target genes (*HES1*, *HEY1*, *HES4*, *p21*) in LoVo cells. The GAPDH promoter serves as a negative control. Data are presented as mean ± SD of three independent experiments. ***P < 0.001, **P < 0.01

**Supplementary Figure S6**

**Figure S6. RBPJ overexpression rescues HP1γ-mediated tumor growth in vivo**

(A) Growth of tumors in nude mice from control, HP1γ overexpression, and HP1γ co-overexpressed with RBPJ LoVo cells (n=7). (B-H) Tumor growth curve of each individual nude mouse. (I) The mean tumor volume change curve of each group of nude mice. Statistical significance analysis was only performed at the final endpoint, and pairwise two-tailed Student’s t-tests were conducted among the three groups. n=7, mean ± SD, ns, no significance; * P<0.05.
